# New species of the genus *Mahinda* Krombein, 1983 (Hymenoptera, Chrysididae, Amiseginae)

**DOI:** 10.3897/zookeys.551.6168

**Published:** 2016-01-11

**Authors:** Lynn S. Kimsey, Toshiharu Mita, Hong Thai Pham

**Affiliations:** 1 Department of Entomology, University of California, One Shields Ave., Davis, California 95616, USA; 2 Entomological Laboratory, Faculty of Agriculture, Kyushu University, Fukuoka 812-8581, JAPAN; 3 Vietnam National Museum of Nature, Vietnam Academy of Science and Technology, Ha Noi, VIETNAM

**Keywords:** Atoposega, Exopapua, Aculeata, Oriental Region

## Abstract

Three new species of *Mahinda* are described, *bo* from Vietnam, *borneensis* from Malaysian Borneo and *sulawesiensis* from northern Sulawesi. A key to the three known species is provided including the previously described species, *saltator* Krombein, 1983.

## Introduction

The genus *Mahinda* Krombein, 1983 includes some of the largest bodied species in the chrysidid subfamily Amiseginae. Females are distinctive, with small pad-like forewings and large, conical or tooth-like propodeal angles. The males associated with *Mahinda saltator* Krombein, 1983, from Sri Lanka are structurally conservative and resemble those of other Asian amisegines, such as male *Myrmecomimesis* Dalla Torre, 1897.

Krombein (1983) associated the male and female of *Mahinda saltator* by shared date and place of collection, for several localities. He made the assumption that they were conspecific since this was the only male amisegine other than males of *Cladobethylus ceylonicus* Krombein, 1980 that was found concurrently with the wingless females. Female *Cladobethylus* are winged. However, the male of *Mahinda saltator* differs substantially from the female in a number of features that are typically shared by both sexes in other genera where sex associations have been made, these differences in *Mahinda saltator* include the presence (female) or absence (male) of an omaulus, the mesopleuron ventromedially simple (females) or grooved and carinate (males), and the presence and number of dorsolongitudinal carinae on the hindcoxa (one in males) or the absence of carinae (females).

**Figure 1.**
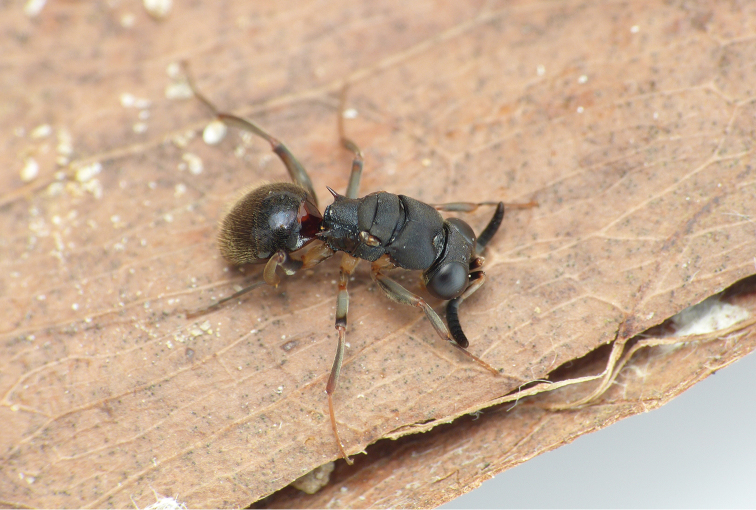
*Mahinda bo* sp. n. live habitus.

The biology of *Mahinda* is unknown but as with other members of the subfamily they are probably parasites of walking stick (Phasmatodea) eggs (Kimsey and Bohart 1991). A female *Mahinda bo* Kimsey, Mita & Pham, sp. n. was observed in the leaf litter in Vietnam by authors Mita and Pham (Fig. 1).

## Materials and methods

Terms used in the descriptions follow those of Kimsey and Bohart (1991). Specimens were studied from the following institutions and/or these are the type repositories:

BMNH
 The Natural History Museum, London, England (Max Barclay);

ROM
Royal Ontario Museum, Edmonton, Ontario, Canada (Chris Darling);

USNM
 U. S. National Museum of Natural History, Washington, D. C., USA (Brian Harris);

VNMN
Vietnam National Museum of Nature, Hanoi, Vietnam.

Specimens were imaged using a Leica video camera mounted on a Leica stereomicroscope (Davis) and Olympus E5 digital camera mounted on an Olympus stereomicroscope (Japan). Images were assembled using the CombineZP software. Line drawings were prepared using a Wild 5 stereomicroscope with drawing tube. The field photo was taken with a digital Panasonic Lumix FZ150 camera, with macro conversion lens (Reynox DCR-250).

## Results

### Genus Mahinda

Taxon classificationAnimaliaHymenopteraChrysididae

Krombein

Mahinda Krombein, 1983:28. Type: *Mahinda saltator* Krombein, 1983:29. Monobasic and original designation

#### Diagnosis.

Female *Mahinda* are brachypterous with the wings reduced to small pads, unlike female *Atoposega*, which are fully winged. They have the lateral propodeal angles sharp and conical, or spine-like and resemble females of *Atoposega* and *Exopapua*. They differ from female *Exopapua* in having a strongly convex pronotum, short metanotum, lack an omaulus and have a gradually sloping propodeum. *Mahinda* females differ from those of *Atoposega* in the hindcoxa with one or no dorsal longitudinal carinae (two in *Atoposega*), mesopleuron with a narrow, parallel-sided ventromedial longitudinal groove (anteriorly carinate and U-shaped in *Atoposega*). In addition *Mahinda* females have two sharp submedial angles above the posterior propodeal declivity, which do not occur in *Atoposega*. Male *Mahinda* have the wings are fully developed and the lateral propodeal angles are not spine-like, but short and conical. Other diagnostic features include the lack of an occipital carina, no omaulus or scrobal sulcus on the mesopleuron, and a long slender stigma + R1. Both sexes have a well-developed malar sulcus.

#### Description.

Head: without occipital carina; eyes covered with short setulae (males) or setulae minute (females); vertical malar sulcus present; propleuron with angulate lateral ridge; scapal basin densely cross-ridged; male flagellum elongate, filiform and cylindrical; female flagellum short and fusiform, flattened on one surface. Mesosoma: pronotum subequal in length to scutum, with short posteromedial groove and pit before lateral lobe; scutum with faint parapsides and well-developed notauli; mesopleuron with (females) or without omaulus (males), without scrobal sulcus, ventromedially flattened with groove and associated U-shaped, carina edged pit (males), convex with simple sulcus or line (females); male fully winged, forewing Rs extended by dark streak in abrupt angle, medial vein arising before cu-a, R1 not clearly indicated, stigma greatly elongate and slender; female strongly brachypterous, forewing pad with several visible veins; metanotum subequal in length to scutellum along midline, with punctate medial enclosure; propodeum with long acute lateral angles in females or short, obtuse angles in males, and abruptly declivous posterior surface; hindcoxa with one (male) or without dorsobasal carina (female); tarsal claw with large medial and small subbasal teeth. Metasoma: integument dull, and densely shagreened, or highly polished and impunctate, vestiture short and decumbent. Sternum I produced into large basal keel.

#### Distribution

**Figure 2.**
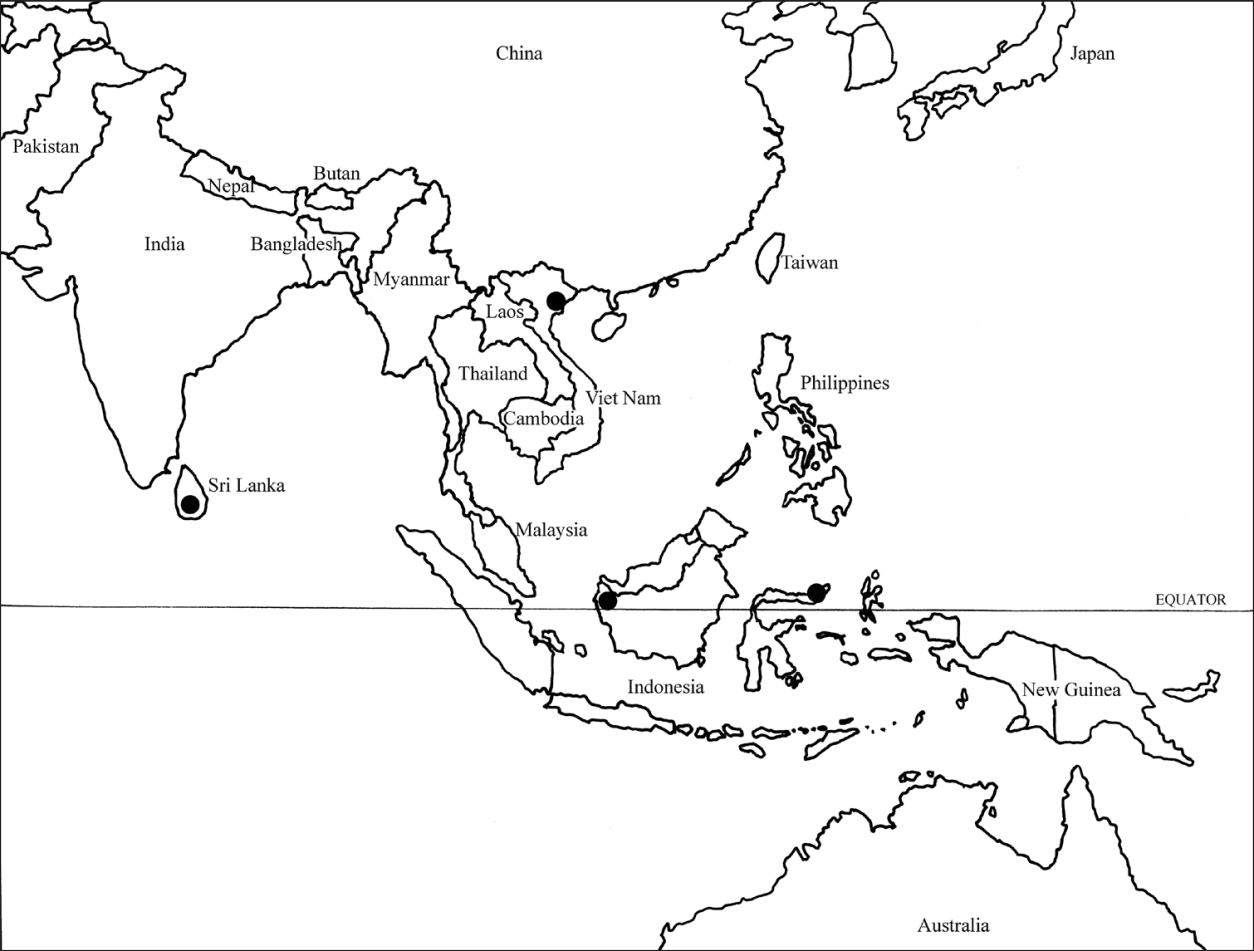
Distribution map of *Mahinda* species.

(Fig. 2). *Mahinda* occurs in Sri Lanka, Vietnam and on the islands of Borneo and Sulawesi. Specimens are rare in collections.

### Key to females of the species of *Mahinda*

**Table:** 

1	Metasomal terga densely, finely shagreened; Sri Lanka	***Mahinda saltator* Krombein**
–	Metasomal terga polished, impunctate or sparsely punctate	**2**
2	Pronotum less than 0.5× as long as broad in dorsal view; propodeal angle long, spike-like, twice or more as long as broad; Vietnam	***Mahinda bo* Kimsey, Mita & Pham, sp. n.**
–	Pronotum more than 0.7× as long as broad in dorsal view; propodeal angle about as broad as long, subtriangular	**3**
3	Mesosoma red (Figs 7, 8); propodeum laterally coarsely cross-ridged; hindocellus separated from nearest eye margin by 1 hindocellar diameter in dorsal view (Fig. 13); Sulawesi	***Mahinda sulawesiensis* Kimsey, Mita & Pham, sp. n.**
–	Mesosoma black (Figs 5, 6); propodeum laterally polished without cross-ridging; hindocellus separated from nearest eye margin by less than 1 hindocellar diameter in dorsal view (Fig. 10); Borneo	***Mahinda borneensis* Kimsey, Mita & Pham, sp. n.**

**Figures 3–8.**
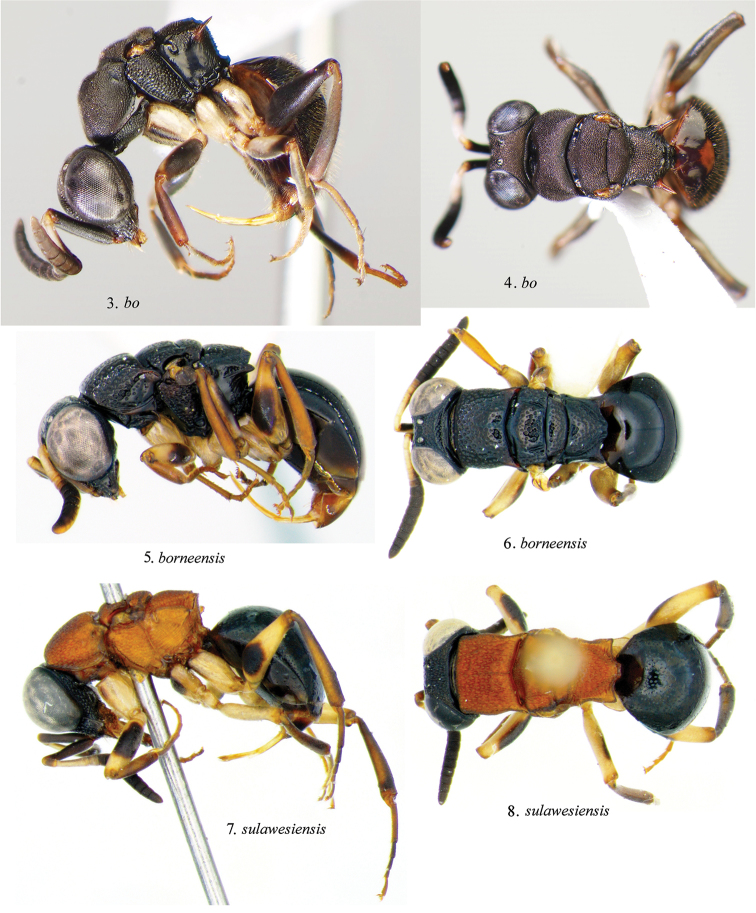
*Mahinda* habitus. **3, 5, 7** Lateral view **4, 6, 8** Dorsal view.

**Figures 9–16.**
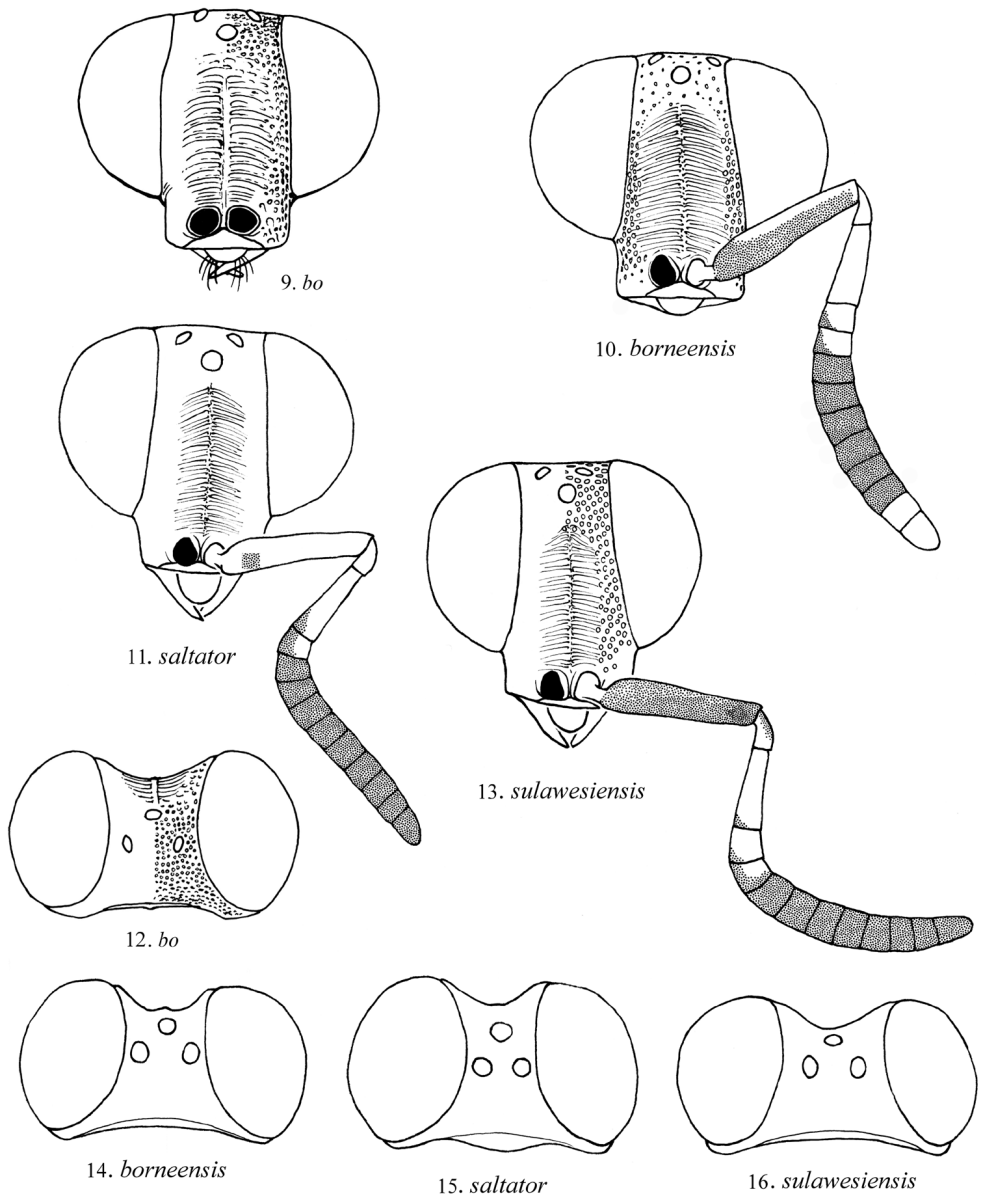
*Mahinda*. **9–11, 13** Front view of face **12, 14–16** Dorsal view of head.

### Mahinda bo

Taxon classificationAnimaliaHymenopteraChrysididae

Kimsey, Mita & Pham sp. n.

http://zoobank.org/64DC013F-5758-4671-8AB8-7612DB30E26E

Figs 1 , 3 , 4 , 9 , 12

#### Diagnosis.

This species can be distinguished from all other species of *Mahinda* by the short, wide, anteromedially indented pronotum and long, spine-like lateral propodeal angles.

#### Female description.

Body length 5.8 mm. Head: face (Fig. 9); scapal basin densely transversely ridged, zone of ridging separated from midocellus by about one midocellar diameter; malar space two midocellar diameters long; subantennal distance 0.5 midocellar diameter long; least interocular distance 0.5× as broad as facial length between midocellus and clypeal apex; eyes slightly converging above in front view; midocellar to ocular margin distance two midocellar diameters; hindocellus one diameter from ocular margin; vertex least interocular distance 0.9× ocular width in dorsal view (Fig. 12); flagellomere I 2.8× as long as broad; flagellomere II 0.7× as long as broad; flagellomere III 0.6× as long as broad; flagellomere XI 0.6× as long as broad; Mesosoma: dorsum and mesopleural punctures separated by 0.5 puncture diameters, polished between; pronotum anteromedially indented, twice as wide as long, 0.7× as long as scutum plus scutellum; metanotum 1.9× as long as broad, with small apicomedial angle on either side of midline; propodeum with lateral tooth long, spike-like, posterior surface coarsely, transversely ridged. Metasoma: terga polished and impunctate. Color (Figs 3, 4): head black, clypeus pale brown medially, mandible dark brown; antenna black except pedicel, flagellomere I and dorsal surface of II whitish; mesosoma black, with pale brown tegula and reddish black lateral propodeal spine; forecoxa whitish, anteriorly black; foretrochanter whitish, ventrally blackish; fore and midfemora black with anterior whitish stripe; tibiae brown, becoming darker laterally; tarsi brown; midcoxa and inner surface of hindfemur entirely whitish; metasoma dark brown becoming blackish dorsally.

#### Male.

Unknown.

#### Type material.

Holotype female: VIETNAM, Bac Giang Province, Tay Yen Tu National park, 21°10'52.33"N 106°43'24.30"E, 176 m, 8/VII/2014, T. Mita leg. (VNMN).

#### Etymology.

The name is derived from the Vietnamese word “bò” for cow, referring to the stout horn-like spines on the propodeum.

### Mahinda borneensis

Taxon classificationAnimaliaHymenopteraChrysididae

Kimsey, Mita & Pham sp. n.

http://zoobank.org/9AA9B1E3-5303-47D0-AD37-AF3418A4D673

Figs 5 , 6 , 10 , 14

#### Diagnosis.

The blackish coloration of the mesosoma and flagellar proportions of *borneensis* most closely resemble those of *saltator*, but the highly polished and impunctate metasoma is most similar to that of *sulawesiensis*. Additionally the posterior and lateral surfaces of the propodeum are smooth without cross-ridging unlike the other species.

#### Female description.

Body length 4.5 mm. Head: face (Fig. 10); scapal basin finely, densely transversely ridged, zone of ridging separated from midocellus by nearly impunctate band more than 1 midocellar diameter wide; malar space 3.4 midocellar diameters long; subantennal distance 0.8 midocellar diameter long; least interocular distance 0.4× as broad as facial length between midocellus and clypeal apex; eyes slightly converging above in front view; midocellar to ocular margin distance 1.8 midocellar diameters; hindocellus 0.5 diameters from ocular margin; vertex least interocular distance 0.8× ocular width in dorsal view (Fig. 14); flagellomere I 2.7× as long as broad; flagellomere II 0.8× as long as broad; flagellomere III 0.7× as long as broad; flagellomere XI 1.4× as long as broad; Mesosoma: dorsum and mesopleural punctures separated by 0.5–1.0 puncture diameters, polished between; pronotum subequal in length to scutum plus scutellum; metanotum 0.6× as long as broad, with small apicomedial angle on either side of midline; propodeum with two posteromedial angles, lateral tooth large, conical, posterior surface polished, with minute punctures near dorsal margin, without medial longitudinal or transverse ridges. Metasoma: terga polished and impunctate. Color (Figs 5, 6): head, mesosoma and metasoma blackish, becoming reddish on mesopleuron and metasomal segments II (apex)-IV and base of tergum I; antenna with scape, pedicel, flagellomere I and parts of II and III whitish, rest of flagellum dark brown; legs pale brown, with darker spot medially on femora.

#### Male.

Unknown.

#### Type material.

Holotype female: Indonesia, Kalimantan, Barat Sungai, Sibau, 21-27/VI/1996, 1°03'N. 113°01'E, 70–90 m, C. Reid, IIS967004 (ROM).

#### Etymology.

The name is derived from Borneo, the island site of collection.

### Mahinda saltator

Taxon classificationAnimaliaHymenopteraChrysididae

Krombein

Figs 11 , 15

Mahinda saltator Krombein 1983a: 29. Holotype female; Sri Lanka: Sabaragamuwa Prov., Kegalla Dist., Kitulgala, Bandarakele Jungle (USNM).

#### Diagnosis.

The finely shagreened metasomal terga are most distinctive feature of this species. The dark mesosomal coloration and flagellomere I 3× or more as long as broad most closely resembles those of *Mahinda borneensis*. Additional diagnostic features include the midocellus 1.3 midocellar diameters from the nearest eye margin, malar space less than 3 midocellus diameters long, thorax with metallic highlights, and face ventrally converging below ocular margins in front view.

#### Female description.

Body length 4.0–4.5 mm. Head: face (Figs 11); scapal basin finely, densely transversely ridged, zone of ridging separated from midocellus by punctate band more than 1 midocellar diameter wide; malar space 2.8 midocellar diameters; subantennal distance 0.9 midocellar diameter long; least interocular distance 0.3× as broad as facial length between midocellus and clypeal apex; eyes convergent above; midocellar to ocular margin distance 1.3 midocellar diameter; hindocellus 0.3–0.4 diameters from ocular margin; vertex least interocular distance 0.7× ocular width in dorsal view (Fig. 15); flagellomere I 2.8× as long as broad; flagellomere II 0.9× as long as broad; flagellomere III 0.7× as long as broad; flagellomere XI 1.5× as long as broad; vertex with dense, contiguous punctures. Mesosoma: dorsum with dense, contiguous punctures, transversely striatiform on pronotum and metanotum, longitudinally striatiform on scutum and scutellum; mesopleural punctures separated by 0.2–0.5 puncture diameters; metanotum 0.6× as long as broad, with small apicomedial angle on either side of midline; propodeum laterally polished, impunctate, posterior surface transversely ridged without medial longitudinal ridge. Metasoma: densely, finely shagreened. Color: head, mesosoma and metasoma black, dorsum of head and mesosoma with faint metallic green to coppery tints; scape red dorsally, brown ventrally; pedicel and flagellomere I yellow, with brown ventroapically; flagellomeres II-III pale brown dorsally, dark brown ventrally; remaining flagellomeres dark brown; coxae yellow, with dark brown patch on anterior surface; femora yellow dorsally, dark brown ventrally; tibiae reddish brown becoming paler basally; tarsi red.

#### Distribution.

This species is only known from the type series of 26 males and 8 females.

### Mahinda sulawesiensis

Taxon classificationAnimaliaHymenopteraChrysididae

Kimsey, Mita & Pham sp. n.

http://zoobank.org/7642030F-F7AC-4A06-9E77-432B58353AF1

Figs 7 , 8 , 13 , 16

#### Diagnosis.

This species most closely resembles *Mahinda borneensis* based on the highly polished metasomal terga and flagellar proportions. *Mahinda sulawesiensis* can be distinguished from *Mahinda borneensis* by the red mesosoma, shorter malar space and broader distance between the hindocellus to the nearest eye margin.

#### Female description.

Body length 5 mm. Head: face (Fig. 13); scapal basin finely, densely transversely ridged, zone of ridging separated from midocellus by punctate band by about 1 midocellar diameter wide; malar space 2.8 midocellar diameters long; subantennal distance 0.6 midocellar diameter long; least interocular distance 0.5× as broad as facial length between midocellus and clypeal apex; midocellar to ocular margin distance 2.5 midocellar diameters in front view; hindocellus 1.4 diameters from ocular margin in dorsal view; vertex least interocular distance equal to ocular width in dorsal view (Fig. 16), punctures coarse, contiguous, striatiform; flagellomere I 3.2× as long as broad; flagellomere II 0.8× as long as broad; flagellomere III 0.7× as long as broad; flagellomere XI 0.6× as long as broad. Mesosoma: dorsum with fine, dense longitudinal scratches among and across punctures; mesopleural punctures large, contiguous; metanotum subtriangular, 0.8× as broad as long, posteromedially bidentate; propodeum laterally with dense longitudinal cross-ridges, posterior surface without medial longitudinal ridge, with medial and sublateral transverse ridging, lateral tooth conical. Metasoma: polished and impunctate. Color (Figs 7, 8): head black, mesosoma red, metasoma black, without metallic highlights; antenna with scape, pedicel, flagellomeres I and part of II and III whitish, rest of flagellum dark brown; legs pale brown with darker medial spot on femora; tarsi brownish.

#### Male.

Unknown.

#### Type material.

Holotype female: Indonesia: Sulawesi, Utara, G. Mogogenipa, 1000 m, VI/1985, A. D. Austin (BMNH).

#### Etymology.

The name is derived from the island of collection.

## Supplementary Material

XML Treatment for Mahinda

XML Treatment for Mahinda bo

XML Treatment for Mahinda borneensis

XML Treatment for Mahinda saltator

XML Treatment for Mahinda sulawesiensis

## References

[B1] Kimsey LS Bohart RM (1991 [“1990”]) The Chrysidid Wasps of the World. Oxford University Press, 652 pp.

[B2] Krombein KV (1983) Biosystematic studies, XI: A monograph of the Amiseginae and Loboscelidiinae. Smithsonian Contributions in Zoology 376: 1–79. doi: 10.5479/si.00810282.376

